# Human autonomy, technological automation (and reverse)

**DOI:** 10.1007/s00146-021-01149-5

**Published:** 2021-02-21

**Authors:** Simona Chiodo

**Affiliations:** Politecnico di Milano, DAStU, Via Bonardi 3, 20133 Milano, Italy

**Keywords:** Autonomy, Automation, Emerging algorithmic technologies, Autonomous vehicles, Anarchism

## Abstract

We continuously talk about autonomous technologies. But how can words qualifying technologies be the very same words chosen by Kant to define what is essentially human, i.e. being autonomous? The article focuses on a possible answer by reflecting upon both etymological and philosophical issues, as well as upon the case of autonomous vehicles. Most interestingly, on the one hand, we have the notion of (human) “autonomy”, meaning that there is a “law” that is “self-given”, and, on the other hand, we have the notion of (technological) “automation”, meaning that there is something “offhand” that is “self-given”. Yet, we are experiencing a kind of twofold shift: on the one hand, the shift from defining technologies in terms of automation to defining technologies in terms of autonomy and, on the other hand, the shift from defining humans in terms of autonomy to defining humans in terms of automation. From a philosophical perspective, the shift may mean that we are trying to escape precisely from what autonomy founds, i.e. individual responsibility of humans that, in the Western culture, have been defined for millennia as rational and moral decision-makers, even when their decisions have been the toughest. More precisely, the shift may mean that we are using technologies, and in particular emerging algorithmic technologies, as scapegoats that bear responsibility for us by making decisions for us. Moreover, if we consider the kind of emerging algorithmic technologies that increasingly surround us, starting from autonomous vehicles, then we may argue that we also seem to create a kind of technological divine that, by being always with us through its immanent omnipresence, omniscience, omnipotence and inscrutability, can always be our technological scapegoat freeing us from the most unbearable burden of individual responsibility resulting from individual autonomy.

## A philosophical question

“Today’s automotive industry relies on three types of vehicle testing: via computer simulation, on real-world public roads, or behind closed doors at a private test track. Approaches vary, but a combination of all three approaches is deemed vital in order to safely introduce highly autonomous vehicles (AVs)”.^1^ This is what we can read in a newspaper almost daily. Surprisingly enough, the *incipit* of the article I have quoted starts and ends with two words that have the same etymology: *autos*, meaning “self”, shared by “automotive” and “autonomous”. Even more surprisingly, in both cases “self” has nothing to do with a human being: “automotive” qualifies “industry” and “autonomous” qualifies “vehicles”, even ending up in an acronym, i.e. “AVs”. If we consider the case of “automotive industry”, then the etymological meaning of *autos* turns out to be fairly ordinary, by identifying something characterised by “self-motion”, and in particular the “industry” that produces “self-moving” objects. On the contrary, if we consider the case of “autonomous vehicles”, then the etymological meaning of *autos* turns out to be fairly extraordinary, by identifying something characterised by a “self-given law”, which is meant by *nomos*, and in particular “vehicles” that have a “self-given law”, and even a “highly” “self-given law”. Thus, the philosophical question cannot but be the following: how can words qualifying “vehicles” be the very same words chosen by Kant to define what is essentially human, i.e. being autonomous?

More precisely, Kant argues that “Autonomy of the will is the property of the will by which it is a law to itself” (Kant 1785: 4: 440). Moreover, being autonomous, i.e. law to oneself, is a kind of culmination of the human being’s evolution, moving from heteronomy to autonomy, the former being “in itself contingent and hence unfit for an apodictic practical rule, such as moral rules must be” (Kant 1785: 4: 444), and the latter being “the ground of the dignity of human nature and of every rational nature” (Kant 1785: 4: 436), founding both freedom (“freedom and the will’s own lawgiving are both autonomy”, Kant 1785: 4: 450) and morality (“For now we see that when we think of ourselves as free we transfer ourselves into the world of understanding as members of it and cognize autonomy of the will along with its consequence, morality”, Kant 1785: 4: 453). Thus, the philosophical question becomes even tougher: how can words qualifying “vehicles” be the very same words chosen by Kant to define human “autonomy of the will”, which founds both human “freedom” and human “morality”?

## An etymological puzzle

If we take a step back searching for an etymological answer, then things do no become less tough, since we find an even more surprising paradox: the word “autonomy”, which is at the very core of the human being’s identity as it is thought of by Kant (among several other philosophers), as well as by several of us, shares the first part of its etymology with the word “automation”, which is at the very core of technology’s identity as we generally think of it, and which is defined by the Oxford English Dictionary as “The action or process of introducing automatic equipment or device into a manufacturing or other process or facility; (also) the fact of making something (as a system, device, etc.) automatic. Originally (and now usually) in neutral sense, but in the 1950s often associated with the use of electronic or mechanical devices to replace human labour, and hence sometimes having negative connotations”.^2^ Thus, paradoxically enough, we have, on the one hand, something that is at the very core of the human being’s identity and, on the other hand, something “to replace” something “human”.

We have to focus on the second part of their etymologies to solve the puzzle. In the case of “autonomy”, as we have seen, we find the ancient Greek noun *nomos*, i.e. “law”. Thus, we obtain a “self-given law”. In the case of “automation”, we find the ancient Greek verb *(auto)matizo*, which is defined by the Liddell-Scott-Jones Greek-English Lexicon as to “act of oneself, act offhand or unadvisedly”, “to be done spontaneously or at random”, “haphazard”, to “introduce the agency of chance”, “of things, [to] happen of themselves, casually”, “to be self-produced” and, “of natural agencies, [to] act spontaneously”.^3^ We also find the ancient Greek noun *automatismos*, which is defined as “that which happens of itself, chance”.^4^ And we also find the ancient Greek noun *automaton*, which, as we shall see, is another word used by Kant, and which is defined as “accident”.^5^ Moreover, *Automatia* is “the goddess of chance”^6^ (see also Murray 1833: 577), who is defined by Smith as a “surname of Tyche or Fortuna, which seems to characterize her as the goddess who manages things according to her own will, without any regard to the merit of man”.^7^ Thus, what do we obtain? The answer is that we obtain what is quite the opposite to a “self-given law”: what is “self-given” has to do with something “offhand”, “unadvised”, “spontaneous”, “random”, “haphazard”, “casual”, “accident[al]” and “without any regard to the merit of man”—on the one hand, we have the notion of “autonomy”, meaning that what is “self-given” is a “law”, and, on the other hand, we have the notion of “automation”, meaning that what is “self-given” is something “offhand”.

If we ascribe the former to humans and the latter to non-humans, and in particular to technologies, then we face the following question: why should we, as “autonomous” humans potentially guided by a “law”, rely on “automated” technologies potentially guided by something “offhand”? Moreover, if we go back to the first issue, then we face a further question, which is the following: how can words qualifying “vehicles”, being “automated”, i.e. potentially guided by something “offhand”, be the very same words qualifying humans, being “autonomous”,^8^ i.e. potentially guided by a “law”?

## From autonomy to morality to culpability

Kant gives us another interesting word to reflect upon: the word “*automaton*”, which shares the etymology of “automation”, but shows two different occurrences. According to Kant, we have an “*automaton materiale*, when the machine is driven by matter” (Kant 1788: 5, 97), and an “*automaton*” “*spirituale*, when it is driven by representations” (Kant 1788: 5, 97). In the latter case, the word “*automaton*” can be ascribed to humans: “if the freedom of our will were none other than the latter (say, psychological and comparative but not also transcendental, i.e. absolute), then it would at bottom be nothing better than the freedom of a turnspit, which, when once it is wound up, also accomplishes its movements of itself” (Kant 1788: 5, 97). Kant argues that, if there is not the kind of autonomy that, as we have seen, founds both freedom and morality, then the human being is nothing but an “*automaton*” “*spirituale*” (whose difference from non-humans, and in particular from things, is nothing but a matter of an adjective, i.e. “*spirituale*”, and not a matter of a noun, i.e. “*automaton*”). And differing from things because of an adjective, and not because of a noun, means dissolving not only autonomy, which is replaced by “*automaton*”, but also its results, i.e. freedom and morality. As for freedom, as we have seen, it dissolves in “the freedom of a turnspit, which, when once it is wound up, also accomplishes its movements of itself”. As for morality, it dissolves even more severely: “no moral law is possible and no imputation in accordance with it” (Kant 1788: 5, 97). Thus, according to Kant, the absence of autonomy means the partial dissolution of freedom and the total dissolution of morality.

Another interesting point made by Kant has specifically to do with the difference between the (total) freedom founded on autonomy and the (partial) freedom founded on heteronomy, i.e. “the freedom of a turnspit”. In the former case, the relationship between the cause of a given action and the action itself is “a free causality” (Kant 1788: 5, 100): the human being’s autonomous will causes its totally free action (which can be moral and, therefore, can be punished if it fails). On the contrary, in the latter case, the relationship between the cause of a given action and the action itself is “the mechanism of nature” (Kant 1788: 5, 97): “all necessity of events in time in accordance with the natural law of causality” (Kant 1788: 5, 97) causes its partially free action (which cannot be moral and, therefore, cannot be punished if it fails). Thus, we obtain a most interesting difference. On the one hand, we find autonomy meaning a kind of (total) freedom that implies morality, which in turn implies, in Kant’s words, “imputation”, as we have seen, as well as being “culpable and deserving of punishment” (Kant 1788: 5, 100). On the other hand, we find heteronomy meaning a kind of (partial) freedom that does not imply morality, which in turn does not imply “imputation”, as well as being “culpable and deserving of punishment”.

I think that we are at a most important point to keep reflecting upon our questions. More precisely, I think that a most important reason why, first, we rely on automated technologies and, second, words qualifying vehicles are the very same words qualifying humans has to do with trying to escape precisely from morality, and in particular from “imputation”, as well as from being “culpable and deserving of punishment”—we try to escape precisely from autonomy, which, by making us free and moral, makes us, at the same time, potentially “culpable and deserving of punishment”.

## Humans as rational and moral decision-makers (for millennia)

If it makes any sense, then we should try to test the shift from automation to autonomy on our technologies, and in particular on emerging algorithmic technologies, which seem to actually replace our autonomy by making decisions for us.

We may say that Kant, as the philosopher who gives us the most powerful lesson on the meaning of autonomy [see at least Korsgaard (1996) and Hill (2000)], teaches us that autonomy means quite the opposite to automation, i.e. something “offhand”, “unadvised”, “spontaneous”, “random”, “haphazard”, “casual”, “accident[al]” and “without any regard to the merit of man”: autonomy is not only, as we have seen, “the property of the will by which it is a law to itself”, but also what founds “all moral concepts [that] have their seat and origin completely a priori in reason, and indeed in the most common reason just as in reason that is speculative in the highest degree; […] they cannot be abstracted from any empirical and therefore merely contingent cognition” (Kant 1785: 4: 440). Thus, speaking of autonomy means speaking of reason—and reason opposes to contingency: if there is autonomy, then there is reason, and, if there is reason, then there is no contingency at all, i.e. nothing “offhand”, “unadvised”, “spontaneous”, “random”, “haphazard”, “casual”, “accident[al]” and “without any regard to the merit of man” at all.

Kant definitely stresses the idea according to which the human being has to be thought of as essentially autonomous and, therefore, characterised by rationality opposing to contingency—the human being is defined as a rational and moral decision-maker, who, first, makes individual decisions rationally and morally and, second, bears the burden of individual responsibility (which means, again, being potentially “culpable and deserving of punishment”).

Anyway, thinking of humans as essentially rational and moral decision-makers is something rooted in the cradle of the European culture. In Plato’s works, we can find an idea of autonomy as rational self-determination leading to morality (especially to human justice) in his description of the tripartite soul, in which rationality rules over the other two irrational parts, which are even described as animals: “‘all our actions and words should tend to give the man within us complete domination over the entire man and make him take charge of the many-headed beast—like a farmer who cherishes and trains the cultivated plants but checks the growth of the wild—and he will make an ally of the lion’s nature, and caring for all the beasts alike will first make them friendly to one another and to himself, and so foster their growth’. ‘Yes, that in turn is precisely the meaning of the man who commends justice’” (Plat. *Resp.* 9, 589 a–b). In Aristotle’s works, we can find an idea of autonomy as rational self-determination leading to morality (especially to human virtue) in his description of the good man, who “work[s] out the good […], and does so for his own sake (for he does it for the sake of the intellectual element in him, which is thought to be the man himself); and he wishes himself to live and be preserved, and especially the element by virtue of which he thinks. […] and the element that thinks would seem to be the individual man, or to be so more than any other element in him” (Arist. *Eth. Nic.* 9, 1166 a 17–23). Moving from ancient philosophy to modern philosophy, we can find not only Kant’s notion of autonomy, as we have seen, but also Rousseau’s work on the importance of autonomy as self-determination in self-development (see especially Rousseau 1762). And, moving from modern philosophy to contemporary philosophy, the stress on autonomy as self-determination even increases, starting from strengthening the notion of individual, which is a typical characteristic of the Western culture. In the nineteenth century, Mill writes that “A person whose desires and impulses are […] not his own has no character, no more than a steam engine has a character” (Mill 1859: 73). It is worth noting that, even if in this passage Mill talks about “desires and impulses”, and not about reason, his metaphor is analogous to Kant’s metaphor: in both cases, absence of autonomy means reduction of the human being to automation, i.e. Kant’s “turnspit” and Mill’s “steam engine”. In the twentieth century, as well as in the last twenty years, autonomy means both the individual exercise of rationality and morality, which is rooted in the cradle of the European culture and definitely developed by Kant, and a kind of procedural individualism freed from the adherence to rational and moral values defined a priori [see especially Dworkin (1988), Frankfurt (1988), Ekstrom (1993) and Bratman (2007)^9^]. In any case, autonomy is crucial when it comes to defining the very core of human identity in the Western culture, especially since the end of the eighteenth century.

Thus, the shift from defining technologies in terms of automation to defining technologies in terms of autonomy seems even more paradoxical: how can we move from ourselves to technologies what has been founding the definition of the very core of our identity for millennia?

## Humans as escapers from autonomy and individual responsibility (today)

It is no coincidence that we can also find the reverse phenomenon, i.e. the shift from defining ourselves in terms of autonomy to defining ourselves in terms of automation. Possible examples are several, sharing the attempt to replace typically human activities founded on autonomy with typically technological activities founded on automation. I shall give at least two examples I surely share with several of us.

The first example is the following. Recently, I happened to see that Google showed wrong information about my academic affiliation. It was embarrassing both for my actual university and for the wrong university. Thus, I informed Google via its online form. But I received an email from a do-not-reply address in which it was said that the information could not be revised. Thus, I proved why the academic affiliation was wrong by providing links to official academic websites proving what the right academic affiliation was. But I received an identical email. I tried again and again. But I received identical emails again and again. Later, I saw that the website of one of my former publishers showed the wrong academic affiliation, which was correct more than a dozen years ago, and which was selected by Google’s algorithm even if several official academic websites showed the right academic affiliation. Thus, I tried again by adding a detailed explanation. But I received an identical email again: paradoxically enough, the most powerful search engine in the world could not show the right information (even if the right information was provided and proved). Later, I myself could find a solution: I asked my former publisher (not via an online form, but by talking to a human being) to revise the wrong academic affiliation, since it changed a dozen years ago. The human being I talked to could both understand and revise the wrong academic affiliation. Later, Google showed the right academic affiliation.

The second example is the following. At several universities, mine included, we use systems to increase automation when it comes to assessing our publications, as one of our criteria to give the departments resources. But we know that, frequently, automation cannot fairly assess exceptions (for instance, articles published on journals listed in the Arts and Humanities Citation Index of Web of Science happened to be assessed as not indexed in Web of Science, since systems were trained to assess scientific and technological articles, which are most of our publications as a technical university’s works. And so forth). Yet, we keep using systems to increase automation when it comes to assessing our publications.

The two examples can show at least one major advantage and one major disadvantage of automation:

1. The advantage (which is the reason why we keep using systems to increase automation when it comes to assessing our publications) is that technology’s automation can do what we used to do both freeing our time from the burden of extended activities and sooner.
2. The disadvantage is that technology’s automation can frequently fail when it comes to exceptions. And exceptions happen frequently. Yet, paradoxically enough, we seem to give priority to automation even over efficiency: in the first example, automation means showing wrong information and, in the second example, automation means assessing exceptions not fairly.

Thus, we should ask why we keep giving priority to automation even over efficiency, and even when efficiency also means fairness. I have been a member of the committee charged with managing the assessment of our publications for years, and I think that we keep giving priority to automation even over efficiency not only because of its major advantage, but also because of something else: we want to have the possibility to say that it is not our fault, but the system’s fault, if something goes wrong—we want to have the possibility to use a technological bureaucracy that may become increasingly opaque, rigid and hard to negotiate with as a way to escape from individual responsibility.

If there is human autonomy, and not technological automation, then both the employee working at Google and the member of the committee have to bear individual responsibility: the former has to face me and my critical requests and the latter has to face their colleagues and their critical requests—moreover, both of them have to face the burden of a possible individual fault.

Thus, both the shift from defining ourselves in terms of autonomy to defining ourselves in terms of automation and the shift from defining technologies in terms of automation to defining technologies in terms of autonomy seem to lead us to a possible reading of our technological era according to which the increasing difficulty in finding who is responsible for something is a symptom of a significant phenomenon: we seem to trade our autonomy for our freedom from individual responsibility—and we seem to find our perfect ally in the kind of technology we are creating, which is helping us escape from autonomy by becoming a scapegoat that bears responsibility, and therefore a kind of autonomy, for us.

## Technology as a scapegoat bearing responsibility for us

Today’s refrain according to which “it is not my responsibility” is exceedingly more than a refrain: it may be one of the distinguishing characteristics of our (technological) era.

We have arrived at the following point: on the one hand, humans seem to take off autonomy and take on a kind of automation and, on the other hand, technology seems to take off automation and take on a kind of autonomy. As for what humans seem to do, we may say that:

1. They seem to take off autonomy by trying to avoid *nomos*, i.e. “law”, at all, in that they are not only searching for a kind of freedom that means overcoming heteronomous “laws” (which was the crucial human evolution Kant worked on), but also searching for a kind of freedom that means overcoming autonomous “laws”, which is precisely what, according to Kant, always implies both being free and being potentially “culpable and deserving of punishment”—autonomous “laws” always imply being individually responsible for what one does.
2. They seem to take on a kind of automation by trying to increase the situations in which, after having moved typically human decision-making processes from themselves to technology, they can say that “it is not my responsibility”. More precisely, they seem to take on a kind of automation in that they are not simply absent at all from decision-making processes: they keep participating in them (in my examples, as the employee working at Google and as the member of the committee), but their role significantly changes—their role significantly moves from bearing individual responsibility for the decision-making process to notifying that the decision-making process is automated. And humans who inescapably keep being responsible for something (in both my examples, for moving decision-making processes from human autonomy to technological automation) become increasingly invisible: I cannot know who is behind the decision not to revise wrong information about my academic affiliation and the colleagues cannot know who is behind the decision to assess an exception in the way it is assessed.

As for what technology seems to do, we may say that:

1. It seems to take off automation at least in that it increasingly assumes definitions typically founded on the notion of autonomy: not only in the case of autonomous vehicles, but also in the cases of increasingly frequent definitions such as autonomous systems, autonomous software, autonomous devices, autonomous applications, autonomous silicon, autonomous things, autonomous machines, autonomous equipment, autonomous drones, autonomous weapons, autonomous robots, autonomous agents, autonomous workloads and so forth.
2. It seems to take on a kind of autonomy at least in that it increasingly assumes tasks typically founded on the notion of autonomy: not only in the case of autonomous vehicles, but also in the case of increasingly frequent tasks, starting from autonomous decision-making.

Most interestingly, rational and moral decisions, which have been what humans had to measure up to in the Western culture for millennia (not only from Plato to Kant to Rawls, but also from Aeschylus to Shakespeare to Pirandello), are becoming a task that is increasingly less human and increasingly more technological—rational and moral decisions as what have been defining individual human merit in the Western culture for millennia are increasingly stopping being an individual’s task.

Even more interestingly, we are increasingly stopping being virtuous. Virtue ethics, which has been one of the cornerstones of morality in the Western culture starting from the ancient Greek philosophy, means that acting in a moral way requires*virtus*, i.e. “virtue”, and in particular, sharing the etymology of *vir*, i.e. “man”, “the sum of all the corporeal or mental excellences of man, *strength*, *vigor; bravery*, *courage; aptness*, *capacity; worth*, *excellence*, *virtue*”,^10^ as well as “*Military talents, courage, valor, bravery, gallantry, fortitude*”.^11^ Thus, being virtuous has meant for millennia the etymological *virilitas*, i.e. “virility”, implying the capacity for bearing one’s own burdens, even when they are severely heavy—being virtuous has meant for millennia the capacity for bearing individual responsibility, even when it is severely heavy, from what one (autonomously) decides to do to what one (autonomously) does (again, being potentially “culpable and deserving of punishment”).

## Our technological era as a radical form of anarchism

Several insights seem to lead us to a possible reading of our technological era as a radical form of anarchism (which is what I tried to argue in Chiodo 2020 extensively). “Anarchism” as the radicalisation of “anarchy” means radical “absence” (*an*) of something that “rules” (*archo*)—“anarchism” means radical “rulerlessness”.

And what else is what “rules” but the “law” meant by *nomos*? Speaking of “anarchism” means removing not only the external “law” implied by heteronomy, but also the internal “law” implied by autonomy. And, if there is no “law” at all, then what remains is strikingly analogous to what is meant by *automatizo* as the etymological meaning of “automation”. It is no coincidence that, from philosophy in particular to culture in general, the authors who more strongly advocate a form of anarchism use words strikingly analogous to what is defined as something “offhand”, “unadvised”, “spontaneous”, “random”, “haphazard”, “casual”, “accident[al]” and “without any regard to the merit of man”, as we have seen. Feyerabend advocates the figure of the epistemological anarchist, according to whom there are no “universal standards, universal laws, universal ideas such as ‘Truth’, ‘Reason’, ‘Justice’, ‘Love’, and the behaviour they bring along” (Feyerabend 1975: 189), since “there is only *one* principle that can be defended […]: anything goes” (Feyerabend 1975: 28). Feyerabend adds that, “like the Dadaist, […] [the epistemological anarchist] ‘not only has no programme, [but also is] against all programmes’” (Feyerabend 1975: 189), and “becomes capable of stepping outside the most fundamental categories and convictions, including those which allegedly make him human” (Feyerabend 1975: 189. See also Rorty 1982, advocating a kind of philosophical anarchist). If we move from philosophy in particular to culture in general, we find Feyerabend’s Dadaist perfectly represented by Tzara, who writes that “I am against systems, the most acceptable system is the one of not having any system, on principle” (Tzara 2001: 299), but “I am also against principles” (Tzara 2001: 300). Thus, any kind of *arche* in general, as well as any kind of *nomos* in particular, is removed. What remains is that “anything goes”. And, if “anything goes”, then there is nothing but something “offhand”, “unadvised”, “spontaneous”, “random”, “haphazard”, “casual”, “accident[al]” and “without any regard to the merit of man”—if “anything goes”, then there is nothing but anarchism.

Interestingly enough, Feyerabend’s epistemological anarchist is described as “stepping outside the most fundamental categories and convictions, including those which allegedly make him human”. We may rhetorically ask what can “allegedly make” someone “human” more than their autonomy as their capacity for making rational and moral decisions, as well as their capacity for bearing the burden of being responsible for them. Less rhetorically, we may ask why someone should remove what “allegedly make[s] him human”. I think that our technological era can make the possible answer clearer than ever.

## A case in point: autonomous vehicles

Let us go back to autonomous technologies. If we read the Waymo Safety Report (*On the road to fully self-driving*), then we find the following sentences: “Waymo’s mission is to bring self-driving technology to the world, making it safe and easy for people and things to move around. We believe our technology can improve mobility by giving people the freedom to get around, and save thousands of lives now lost to traffic crashes” (Waymo Safety Report 2017: 2); “Our ultimate goal is to develop fully self-driving technology that can take someone from A to B, anytime, anywhere, and in all conditions” (Waymo Safety Report 2017: 16); “During our internal testing, however, we found that human drivers over-trusted the technology [Level 3] and were not monitoring the roadway carefully enough to be able to safely take control when needed. As driver-assist features become more advanced, drivers are often asked to transition from passenger to driver in a matter of seconds, often in challenging or complex situations with little context of the scene ahead. The more tasks the vehicle is responsible for, the more complicated and vulnerable this moment of transition becomes. Avoiding this ‘handoff problem’ is part of the reason why Waymo is working on fully self-driving vehicles. Our technology takes care of all of the driving, allowing passengers to stay passengers” (Waymo Safety Report 2017: 13). First, it is worth noting that rhetoric stresses the idea of discharging humans from responsibility: if you are a passenger of an autonomous vehicle, then you are “free”, since something else takes you “from A to B, anytime, anywhere, and in all condition” in a “safe and easy” way. Second, it is worth noting something paradoxical: since humans seem to escape from responsibility by relying on technology even excessively (“human drivers over-trusted the technology [Level 3] and were not monitoring the roadway carefully enough to be able to safely take control when needed”), the solution is not to ask humans to take on more responsibility, but to ask humans not to take on responsibility at all (“Waymo is working on fully self-driving vehicles […] allowing passengers to stay passengers”, i.e. allowing passive humans to stay passive humans). I do not deny at all the potential advantages of autonomous vehicles, from decreasing collisions to energy saving to increasing inclusiveness for the benefit of the elderly, for instance. But what a philosopher should do is trying to understand the inner meaning of a phenomenon—and I think that the inner meaning of autonomous technologies has to do with desperately trying to reverse the roles by making ourselves increasingly less autonomous and technology increasingly more autonomous.

It is no coincidence that the image of the paragraph *The case for full autonomy: allowing passengers to stay passengers* (Waymo Safety Report 2017: 13) significantly coincides with two human hands leaving the wheel, i.e. the symbol of human control (human hands, such as when we even idiomatically say to have the situation in hand) leaving the symbol of control (the wheel, such as when we even idiomatically say to be behind the wheel).

Moreover, in theWaymo website we can read that “Fully self-driving vehicles hold the promise to improve road safety”,^12^ which may be true. But, again, what a philosopher should do is trying to understand the inner meaning of a phenomenon—and the words describing the phenomenon are exceedingly revealing: one of the most typically human prerogatives, i.e. “hold[ing] the promise”, is exercised not by humans, but by technology: technology is even described as what can always “hold the promise” that humans cannot always “hold”.

Thus, we find again the kind of twofold shift we have seen: on the one hand, the shift from defining technologies in terms of automation to defining technologies in terms of autonomy (which can even make them capable of “hold[ing] the promise”) and, on the other hand, the shift from defining ourselves in terms of autonomy to defining ourselves in terms of automation (which can even make us as passive as “passengers” who “stay passengers” “anytime, anywhere, and in all conditions” without “monitoring”, “tak[ing] control” and being “responsible for” “tasks”).

If it makes any sense, then the possible answer to the question on why someone should remove what “allegedly make[s] him human” becomes clearer—the point may be that what makes us human the most is precisely our most unbearable burden, i.e. the burden of individual responsibility, which potentially makes us not only “culpable and deserving of punishment”, but also a failure.

## The most radical form of anarchism we have ever experienced

Yet, for the first time in human history, we can overcome the burden of individual responsibility through technology, by increasingly discharging ourselves from what can make us both “culpable and deserving of punishment” and a failure the most: individual decision-making.

I think that the inner meaning of the phenomenon described can be read as the most radical form of anarchism we have ever experienced. And it may frequently happen that, if there is the risk of falling into a form of extremism, then there is also the risk of falling into its opposite form of extremism, which is totalitarianism (even if, as I shall try to explain, it is significantly different from the form of totalitarianism we have experienced in the twentieth century).

As for the most radical form of anarchism we have ever experienced, I may try to summarise my arguments (see again Chiodo 2020) as follows:

1. First, as we have seen, we use technology to remove the kind of law that is the hardest law to remove: the internal law (even if it is precisely what founds our autonomy). I may add to what we have seen at least a last example: anytime we google something, we give a private technological company the power to become the verb itself that replaces our individual capacity for deciding through our imagination and thought, for instance, what to eat today (since our “over-trust[ing] the technology” makes us usually stop at the first webpage, which shows us no more than a dozen possibilities selected by an algorithm). What happens if we replace our individual capacity for deciding through our imagination and thought not only what to eat today, but also who to vote for tomorrow? In this case, anarchism shows up precisely as what its etymology means: if we do not have any kind of *arche* in general, as well as any kind of *nomos* in particular, then we have nothing but radical “rulerlessness”—and our votes, which are exceedingly important decisions, will be nothing but something “offhand”, “unadvised”, “spontaneous”, “random”, “haphazard”, “casual”, “accident[al]” and “without any regard to the merit of man” (unfortunately, this is not only a hypothetical scenario).
2. Second, we use technology to remove the role of the expert as a mediator, which is a way to remove the external law. Yet, it is not always wise to remove the external law, at least as an external knowledge that our internal decision reflectively considers. But we use technology to remove the role of the expert as a mediator again and again—we use technology as a kind of do-it-yourself *passe-partout* replacing any kind of authentic expertise. This is precisely what happens, for instance, anytime we take our smartphone, google our symptom and self-diagnose. In this case, anarchism shows up in that we self-diagnose without being doctors (and even quarrel with doctors if their diagnosis differs from ours). The expert as a mediator, i.e. the doctor, is literally replaced by the immediacy of our googling our symptom and self-diagnose. And the *arche*, as well as the *nomos*, i.e. the authentic expertise that can guide us, dissolves as the difference between our capacity for self-diagnosing through googling and the doctor’s capacity for diagnosing dissolves—and, again, our self-diagnosis will be nothing but something “offhand”, “unadvised”, “spontaneous”, “random”, “haphazard”, “casual”, “accident[al]” and “without any regard to the merit of man”. To add at least a last example, this is precisely what happens, for instance, in the case of populist parties such as the Italian Five Star Movement, in whose website we can read that it works “outside associative and party ties and without the mediation of governing or representative bodies, recognizing to all citizens the governing and steering role normally attributed to few”,^13^ which means not only the risk of electing incompetent citizens, but also the use of technology (the digital platform Rousseau^14^) to launch a kind of e-democracy that makes activists vote in complex referenda and contribute to drafting complex laws even if they lack any professional competence. Again, the role of the expert as a mediator, in this case between people’s incompetence and politicians’ competence, is removed^15^—and, again, removing the role of the expert as a mediator means making people “rulerless”, i.e., when voting in complex referenda and contributing to drafting complex laws, pushed by contingent opinions, i.e. *doxa*,^16^ which are nothing but something “offhand”, “unadvised”, “spontaneous”, “random”, “haphazard”, “casual”, “accident[al]” and “without any regard to the merit of man”.^17^
3. Third, and moreover, we use technology to try, for the first time in human history, to replace the role of a transcendent divine itself by creating, especially through information technology, a totally immanent technological entity characterized by the typical ontological prerogatives of the divine: omnipresence (by being everywhere), omniscience (by knowing everything, especially about us), omnipotence (by having power, especially over us) and inscrutability (by being frequently founded on algorithms that are black boxes)—and making the divine immanent may be thought of as the most radical form of anarchism we have ever experienced. Anytime we take our smartphone, google our symptom and self-diagnose, we use a technology that is, on the one hand, omnipresent, omniscient and omnipotent and, on the other hand, not transcendent at all, but totally immanent, being a human creation. And the ultimate result of being a totally immanent human creation is that, for instance, the kind of knowledge it gives us is outwardly perfect, but inwardly characterised by the typical ontological prerogatives of the human: imperfection, i.e., again, nothing but something “offhand”, “unadvised”, “spontaneous”, “random”, “haphazard”, “casual”, “accident[al]” and “without any regard to the merit of man” (it is no coincidence that, if we google our symptom today and tomorrow, we may happen to find different results, which are characterised, therefore, by contingency: the kind of truth we may happen to obtain is nothing but something as contingent as an anarchist kind of truth). Yet, we keep creating a totally immanent technological divine, which can give us the great advantage we are increasingly interested in: externalising our decision-making processes, i.e. creating a totally immanent technological divine that can make epistemological and ethical decisions for us. If it makes any sense, then we can define our technological era as the most radical form of anarchism we have ever experienced in that we are technologically creating a kind of divine that is actually the greatest *automaton materiale*—and more precisely the greatest *automaton materiale* that, by being always with us through its immanent omnipresence, omniscience, omnipotence and inscrutability, can always be our technological scapegoat freeing us from the most unbearable burden of individual responsibility resulting from individual autonomy.

## The (novel form of) totalitarian risk

As for the risk of falling into a form of totalitarianism as its opposite form of extremism, we should not forget that, anyway, the form of totalitarianism we risk falling into today is novel, i.e. notably different from the form of totalitarianism we have experienced in the twentieth century. In the latter case, from an epistemological perspective, we tried to make the ideal real. More precisely, the ideal was the vision of the perfect counterpart of reality (from ideal moral visions to ideal political visions), and we tried to make the ideal vision real, even if, as Plato teaches us in his *Republic*, it is not possible at all. Thus, any attempt to make the ideal real resulted in a dramatic failure (see especially Berlin 1990 and 2006).

On the contrary, the form of totalitarianism we risk falling into today is not an attempt to make the ideal real at all—it is quite the opposite: again, it results from anarchism. Anytime we risk, for instance, being hacked by a technology company, as Harari would argue, i.e. being manipulated when it comes, for instance, to decide what to buy, it is not a matter of the technology company’s attempt to make the ideal real—on the contrary, it is a matter of contingently making more money precisely by taking advantage of the absence of ideals. If we have a ruling *arche*, which may be, for instance, an ideal vision of what we need and why, as well as an ideal vision of what balance consumption is, then it is harder for the technology company to make us buy anything it has the contingent economic interest to make us buy. But, if we have no ruling *arche* at all, i.e. no ideal vision at all, then we are easy prey for a form of totalitarianism that has nothing to do with making ideal visions real, resulting, on the contrary, from filling the void of ideal visions with whoever’s contingent interest—the more we are anarchist (in the authentic sense of the word), the easier we are prey for making whoever’s contingent interest win out over us.

After all, the phenomenon I have described may be the natural consequence of the kind of twofold shift we have seen: on the one hand, the more we define technologies in terms of autonomy, the more they may be the means by which whoever’s contingent interest can win out over us and, on the other hand, the more we define ourselves in terms of automation, the easier prey we may become for whoever’s contingent interest—not only in that we are increasingly giving away our autonomy to technologies, but also, and moreover, in that, by stopping exercising our autonomy to make decisions, we are increasingly stopping exercising what it essentially means, i.e. our rationality and morality.

Thus, technology seems to increasingly take on prerogatives from any ontological dimension. As we have seen, it is taking on human prerogatives by obtaining a kind of autonomy—and more precisely the kind of autonomy that seems to have become our most unbearable burden (which has become even more unbearable as our society has become unbearably competitive in the last years). But it is also taking on divine prerogatives by obtaining a kind of omnipresence, omniscience, omnipotence and inscrutability. We may keep doing what we are doing. Yet, we should at least reflect upon what follows: anytime we trade our autonomy for our freedom from individual responsibility, we not only win a technological scapegoat, so that we can say “it’s not our fault!”, but also lose the very core of our identity as it has been thought of in the Western culture for millennia, i.e. as rational and moral decision-makers—as autonomous humans. And losing our autonomy as our capacity for making rational and moral decisions may mean losing precisely what exercises our most essential capacity: our capacity for evolving, by being continuously pushed by puzzling rational and moral challenges—and it is no coincidence that, again, as we may be losing our capacity for evolving, technology may be winning it, proving day by day to be what is becoming the most capable when it comes to strikingly evolving, and strikingly quickly.

## Footnotes

1 See https://www.automotiveworld.com/articles/private-test-tracks-are-where-autonomous-vehicles-drive-risk-free/.

2 See https://www.oed.com/view/Entry/13468?redirectedFrom=automation#eid.

3 See http://stephanus.tlg.uci.edu/lsj/#eid=18225.

4 See http://stephanus.tlg.uci.edu/lsj/#eid=18225.

5 See http://stephanus.tlg.uci.edu/lsj/#eid=18228.

6 See http://stephanus.tlg.uci.edu/lsj/#eid=18224.

7 Quote from Smith 1867, available online: https://quod.lib.umich.edu/m/moa/acl3129.0001.001/462?page=root;rgn=full+text;size=100;view=image;q1=auto.

8 Interestingly enough, Europe seems to make the word “automated” win out over the word “autonomous” when it comes to defining AVs. See especially the recent recommendations by the Horizon 2020 Commission Expert Group to advise on specific ethical issues raised by driverless mobility: *Ethics of connected and automated vehicles. Recommendations on road safety, privacy, fairness, explainability and responsibility*. See also the German report of the Federal Minister of Transport and Digital Infrastructure, *Ethics Commission. Automated and connected driving*, and the European Technology Platform on Smart Systems Integration *European roadmap. Smart systems for automated driving*.

9 For an overview, see also https://iep.utm.edu/autonomy/.

10 See http://www.perseus.tufts.edu/hopper/morph?l=virtus&la=la&can=virtus0#lexicon.

11 See http://www.perseus.tufts.edu/hopper/morph?l=virtus&la=la&can=virtus0#lexicon.

12 See https://waymo.com/.

13 See https://www.movimento5stelle.it/ (my translation).

14 See https://vote.rousseau.movimento5stelle.it/.

15 It is no coincidence that their political leader from 2017 to 2020, without both a degree and a consolidated professional expertise, was the Minister of Economic Development, as well as the Minister of Labour and Social Policy, in 2018 (at the age of thirty-two) and the Minister of Foreign Affairs and International Cooperation in 2019 (at. the age of thirty-three).

16 Meaning, from an epistemological perspective, the “subjective knowledge” that, being particular, contingent, and uncertain, opposes to *episteme*, meaning, from an epistemological perspective, the “objective knowledge” that is universal, absolute, and certain.

17 Again, it is no coincidence that there is an interesting correlation between the Five Star Movement and one of the most typical consequences of epistemological anarchism: the risk to fall into the phenomenon of fake news. According to the *New York Times* editorial *Populism, politics, and measles* (May 2, 2017), “In Italy, the populist Five Star Movement (M5S) led by the comedian Beppe Grillo has campaigned actively on an anti-vaccination platform, likewise repeating the false ties between vaccinations and autism” (which are words that sound shocking as we face the coronavirus emergency).
